# Complete genome sequence of *Brachyspira murdochii* type strain (56-150^T^)

**DOI:** 10.4056/sigs.831993

**Published:** 2010-06-15

**Authors:** Amrita Pati, Johannes Sikorski, Sabine Gronow, Christine Munk, Alla Lapidus, Alex Copeland, Tijana Glavina Del Tio, Matt Nolan, Susan Lucas, Feng Chen, Hope Tice, Jan-Fang Cheng, Cliff Han, John C. Detter, David Bruce, Roxanne Tapia, Lynne Goodwin, Sam Pitluck, Konstantinos Liolios, Natalia Ivanova, Konstantinos Mavromatis, Natalia Mikhailova, Amy Chen, Krishna Palaniappan, Miriam Land, Loren Hauser, Yun-Juan Chang, Cynthia D. Jeffries, Stefan Spring, Manfred Rohde, Markus Göker, James Bristow, Jonathan A. Eisen, Victor Markowitz, Philip Hugenholtz, Nikos C. Kyrpides, Hans-Peter Klenk

**Affiliations:** 1 DOE Joint Genome Institute, Walnut Creek, California, USA; 2 DSMZ – German Collection of Microorganisms and Cell Cultures GmbH, Braunschweig, Germany; 3 Los Alamos National Laboratory, Bioscience Division, Los Alamos, New Mexico, USA; 4 Biological Data Management and Technology Center, Lawrence Berkeley National Laboratory, Berkeley, California, USA; 5 Oak Ridge National Laboratory, Oak Ridge, Tennessee, USA; 6 HZI – Helmholtz Centre for Infection Research, Braunschweig, Germany; 7 University of California Davis Genome Center, Davis, California, USA

**Keywords:** host-associated, non-pathogenic, motile, anaerobic, Gram-negative, *Brachyspiraceae*, *Spirochaetes*, GEBA

## Abstract

*Brachyspira murdochii* Stanton *et al*. 1992 is a non-pathogenic, host-associated spirochete of the family *Brachyspiraceae*. Initially isolated from the intestinal content of a healthy swine, the ‘group B spirochaetes’ were first described as *Serpulina murdochii*. Members of the family *Brachyspiraceae* are of great phylogenetic interest because of the extremely isolated location of this family within the phylum ‘*Spirochaetes*’. Here we describe the features of this organism, together with the complete genome sequence and annotation. This is the first completed genome sequence of a type strain of a member of the family *Brachyspiraceae* and only the second genome sequence from a member of the genus *Brachyspira*. The 3,241,804 bp long genome with its 2,893 protein-coding and 40 RNA genes is a part of the *** G****enomic* *** E****ncyclopedia of* *** B****acteria and* *** A****rchaea * project.

## Introduction

Strain 56-150^T^ (= DSM 12563 = ATCC 51284 = CIP 105832) is the type strain of the species *Brachyspira murdochii*. This strain was first described as *Serpulina murdochii* [1,2], and later transferred to the genus *Brachyspira* [3]. The genus *Brachyspira* currently consists of seven species, with *Brachyspira aalborgi* as the type species [4,5]. The genus *Brachyspira* is the only genus in the not yet formally described family ‘*Brachyspiraceae*’ [6,7]. The generic name derives from ‘brachys’, Greek for short, and ‘spira’, Latin for a coil, a helix, to mean ‘a short helix’ [5]. The species name for *B. murdochii* derives from the city of Murdoch, in recognition of work conducted at Murdoch University in Western Australia, where the type strain was identified [1]. Some species of the genus *Brachyspira* cause swine dysentery and porcine intestinal spirochetosis. Swine dysentery is a severe, mucohemorrhagic disease that sometimes leads to death of the animals [1]. *B. murdochii* is generally not considered to be a pathogen, although occasionally it has been seen in association with colitis in pigs [3,8], and was also associated with clinical problems on certain farms [9-11].

In 1992, a user-friendly and robust novel PCR-based restriction fragment length polymorphism analysis of the *Brachyspira* *nox*-gene was developed, which allows one to identify, with high specificity, members of *B. murdochii* using only two restriction endonucleases [12]. More recently, a multi-locus sequence typing scheme was developed that facilitates the identification of *Brachyspira* species and reveals the intraspecies diversity of *B. murdochii* [13] (see also http://pubmlst.org/brachyspira/).

Only one genome of a member of the family ‘*Brachyspiraceae*’ been sequenced to date: *B. hyodysenteriae* strain WA1 [14],. It is an intestinal pathogen of pigs. Based on 16S rRNA sequence this strain is 0.8% different from strain 56-150^T^. Here we present a summary classification and a set of features for *B. murdochii* 56-150^T^, together with the description of the complete genomic sequencing and annotation.

## Classification and features

*Brachyspira* species colonize the lower intestinal tract (cecum and colons) of animals and humans [6]. The type of *B. murdochii*, 56-150^T^, was isolated from a healthy swine in Canada [1,15]. Other isolates have been obtained from wild rats in Ohio, USA, from laboratory rats in Murdoch, Western Australia [16], and from the joint fluid of a lame pig [17]. Further isolates have been obtained from the feces or gastrointestinal tract of pigs in Canada, Tasmania, Queensland, and Western Australia [2,15]. The type strains of the other species of the genus *Brachyspira* share 95.9-99.4% 16S rRNA sequence identity with strain 56-150^T^. GenBank contains 16S rRNA sequences for about 250 *Brachyspira* isolates, all of which share at least 96% sequence identity with strain 56-150^T^ [18]. The closest related type strain of a species outside of the *Brachyspira,* but within the order *Spirochaetales,* is *Turneriella parva* [19], which exhibits only 75% 16S rRNA sequence similarity [18]. 16S rRNA sequences from environmental samples and metagenomic surveys do not exceed 78-79% sequence similarity to strain 56-150^T^, with the sole exception of one clone from a metagenome analysis of human diarrhea [20], indicating that members of the species, genus and even family are poorly represented in the habitats outside of various animal intestines screened thus far (status March 2010).

**Figure 1:**
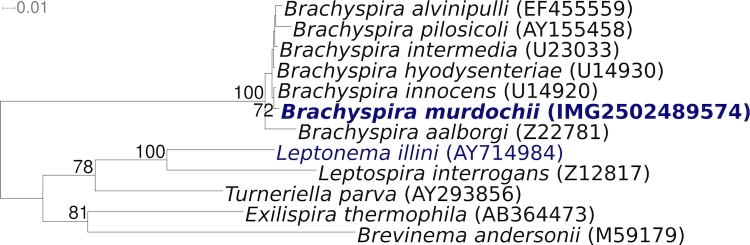
Phylogenetic tree highlighting the position of *B. murdochii* 56-150^T^ relative to the other type strains within the genus and to the type strains of the other genera within the class *Spirochaetes* (excluding members of the *Spirochaetaceae*)*.* The tree was inferred from 1,396 aligned characters [21,22] of the 16S rRNA gene sequence under the maximum likelihood criterion [23] and rooted in accordance with the current taxonomy. The branches are scaled in terms of the expected number of substitutions per site. Numbers above branches are support values from 1,000 bootstrap replicates if [24] larger than 60%. Lineages with type strain genome sequencing projects registered in GOLD [25] are shown in blue, published genomes in bold.

Figure 1 shows the phylogenetic neighborhood of *B. murdochii* 56-150^T^ in a 16S rRNA based tree. The sequence of the single 16S rRNA gene in the genome sequence is identical with the previously published 16S rRNA gene sequence generated from DSM 12563 (AY312492).

**Figure 2:**
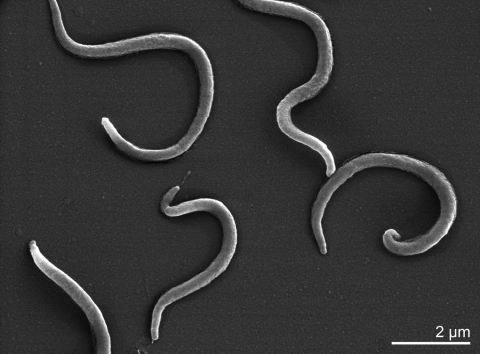
Scanning electron micrograph of *B. murdochii* 56-150^T^

The cells of *B. murdochii* 56-l50^T^ were 5 - 8 by 0.35 - 0.4 µm in size (Table 1 and Figure 2), and each cell possessed 22 to 26 flagella (11 to 13 inserted at each end) [1]. In brain/heart infusion broth containing 10% calf serum (BHIS) under an N_2_-O_2_ (99::l) atmosphere, strain 56-150^T^ had optimum growth temperatures of 39 to 42°C (shortest population doubling times and highest final population densities) [1]. In BHIS broth at 39°C, the doubling times of strain 56-150^T^ were 2 to 4 h, and the final population densities were 0.5 x l0^9^ to 2.0 x l0^9^ cells/ml. Strain 56-150^T^ did not grow at 32 or 47°C [1].

**Table 1:** Classification and general features of *B. murdochii* 56-150^T^  according to the MIGS recommendations [26]

**MIGS ID**	**Property**	**Term**	**Evidence code**
	Current classification	Domain *Bacteria*	TAS [27]
		Phylum *Spirochaetes*	TAS [28]
		Class *Spirochaetes*	TAS [28]
		Order *Spirochaetales*	TAS [29,30]
		Family *Brachyspiraceae*	TAS [31]
		Genus *Brachyspira*	TAS [5]
		Species *Brachyspira murdochii*	TAS [1]
		Type strain 56-150	TAS [1]
	Gram stain	negative	TAS [1]
	Cell shape	helical cells with regular coiling pattern	TAS [1]
	Motility	motile (periplasmic flagella)	TAS [1]
	Sporulation	non-sporulating	TAS [1]
	Temperature range	does not grow at 32°C or 47°C	TAS [1]
	Optimum temperature	39°C	TAS [1]
	Salinity	unknown	TAS
MIGS-22	Oxygen requirement	anaerobic, aerotolerant	TAS [1]
	Carbon source	soluble sugars	TAS [1]
	Energy source	chemoorganotrophic	TAS [1]
MIGS-6	Habitat	animal intestinal tract	TAS [6]
MIGS-15	Biotic relationship	host-associated	TAS [32]
MIGS-14	Pathogenicity	no	TAS [33]
	Biosafety level	1	TAS [34]
	Isolation	swine	TAS [15]
MIGS-4	Geographic location	Quebec, Canada	TAS [15]
MIGS-5	Sample collection time	1992	TAS [15]
MIGS-4.1 MIGS-4.2	Latitude Longitude	52.939 -73.549	TAS [1] TAS [1]
MIGS-4.3	Depth	not reported	TAS
MIGS-4.4	Altitude	not reported	TAS

Evidence codes - IDA: Inferred from Direct Assay (first time in publication); TAS: Traceable Author Statement (i.e., a direct report exists in the literature); NAS: Non-traceable Author Statement (i.e., not directly observed for the living, isolated sample, but based on a generally accepted property for the species, or anecdotal evidence). These evidence codes are from of the Gene Ontology project [35]. If the evidence code is IDA, then the property was directly observed by one of the authors or an expert mentioned in the acknowledgements

Substrates that support growth of strain 56-150^T^ in HS broth (basal heart infusion broth containing 10% fetal calf serum) include glucose, fructose, sucrose, N-acetylglucosamine, pyruvate, L-fucose, cellobiose, trehalose, maltose, mannose, and lactose, but not galactose, D-fucose, glucosamine, ribose, raffinose, rhamnose, or xylose [1]. In HS broth supplemented with 0.4% glucose under an N_2_-O_2_ (99:l) atmosphere, the metabolic end products of strain 56-150^T^ are acetate, butyrate, ethanol, CO_2_, and H_2_. Strain 56-150^T^ produces more H_2_ than CO_2_ [1], which is indicative of NADH-ferredoxin oxidoreductase reaction [6]. The ethanol is likely to be formed from acetyl-CoA by the enzymes acetaldehyde dehydrogenase and alcohol dehydrogenase [6]. Strain 56-150^T^ is weakly hemolytic, negative for indole production, does not hydrolyze hippurate, is negative for α-galactosidase and α-glucosidase activity, but positive for β-glucosidase activity [1]. Strain 56-150^T^ is anaerobic but aerotolerant [1].

Minimal inhibitory concentrations have been determined for strain 56-150^T^ for tiamulin hydrogen fumarate, tylosin tartrate, erythromycin, clindamycin hydrochloride, virginiamycin, and carbadox [36]. Several strains of *B. murdochii* have been described to be naturally resistant against the rifampicin [7,32]. Also, a ring test for quality assessment for diagnostics and antimicrobial susceptibility testing of the genus *Brachyspira* has been reported [37].

### Chemotaxonomy

At present there are no reports on the chemotaxonomy of *B. murdochii*. However, some data are available for *B. innocens* (formerly classified as *Treponema innocens* [6]), the species that is currently most closely related to *B. murdochii* [13]. *B. innocens* cellular phospholipids and glycolipids were found to contain acyl (fatty acids with ester linkage) with alkenyl (unsaturated alcohol with ether linkage) side chains [6,38]. The glycolipid of *B. innocens* contains monoglycosyldiglyceride (MGDG) and, in most strains, acylMGDG is also found, with galactose as the predominant sugar moiety [38].

## Genome sequencing and annotation

### Genome project history

This organism was selected for sequencing on the basis of its phylogenetic position [39], and is part of the *** G****enomic* *** E****ncyclopedia of* *** B****acteria and* *** A****rchaea * project [40]. The genome project is deposited in the Genome OnLine Database [25] and the complete genome sequence is deposited in GenBank Sequencing, finishing and annotation were performed by the DOE Joint Genome Institute (JGI). A summary of the project information is shown in Table 2.

**Table 2:** Genome sequencing project information

**MIGS ID**	**Property**	**Term**
MIGS-31	Finishing quality	Finished
MIGS-28	Libraries used	Four genomic libraries: two Sanger 6kb and 8 kb pMCL200 library, one fosmid library, one 454 standard library
MIGS-29	Sequencing platforms	ABI3730, 454 GS FLX
MIGS-31.2	Sequencing coverage	19.7× Sanger; 48.9× pyrosequence
MIGS-30	Assemblers	Newbler version 1.1.02.15, phrap
MIGS-32	Gene calling method	Prodigal 1.4, GenePRIMP
	INSDC ID	CP001959
	Genbank Date of Release	May 13, 2010
	GOLD ID	Gc01276
	NCBI project ID	29543
	Database: IMG-GEBA	2502422316
MIGS-13	Source material identifier	DSM 12563
	Project relevance	Tree of Life, GEBA

### Growth conditions and DNA isolation

*B. murdochii,* strain 56-150^T^, DSM 12563, was grown anaerobically in DSMZ medium 840 (*Serpulina murdochii* medium) [41] at 37°C. DNA was isolated from 0.5-1 g of cell paste using Qiagen Genomic 500 DNA Kit (Qiagen, Hilden, Germany) with lysis modification st/L according to Wu *et al*. [40].

### Genome sequencing and assembly

The genome was sequenced using a combination of Sanger and 454 sequencing platforms. All general aspects of library construction and sequencing performed can be found at the JGI website (http://www.jgi.doe.gov/). In total, 861,386 Pyrosequencing reads were assembled using the Newbler assembler version 1.1.02.15 (Roche). Large Newbler contigs were broken into 3,554 overlapping fragments of 1,000 bp and entered into assembly as pseudo-reads. The sequences were assigned quality scores based on Newbler consensus q-scores with modifications to account for overlap redundancy and adjust inflated q-scores. A hybrid 454/Sanger assembly was made using the parallel phrap assembler (High Performance Software, LLC). Possible misassemblies were corrected with Dupfinisher or transposon bombing of bridging clones [42]. A total of 300 Sanger finishing reads were produced to close gaps, to resolve repetitive regions, and to raise the quality of the finished sequence. The error rate of the completed genome sequence is less than 1 in 100,000. Together, the combination of the Sanger and 454 sequencing platforms provided 68.6× coverage of the genome. The final assembly contains 79,829 Sanger reads and 861,386 pyrosequencing reads.

### Genome annotation

Genes were identified using Prodigal [43] as part of the Oak Ridge National Laboratory genome annotation pipeline, followed by a round of manual curation using the JGI GenePRIMP pipeline [44]. The predicted CDSs were translated and used to search the National Center for Biotechnology Information (NCBI) nonredundant database, UniProt, TIGR-Fam, Pfam, PRIAM, KEGG, COG, and InterPro databases. Additional gene prediction analysis and functional annotation was performed within the Integrated Microbial Genomes - Expert Review (IMG-ER) platform [45].

## Genome properties

**Figure 3:**
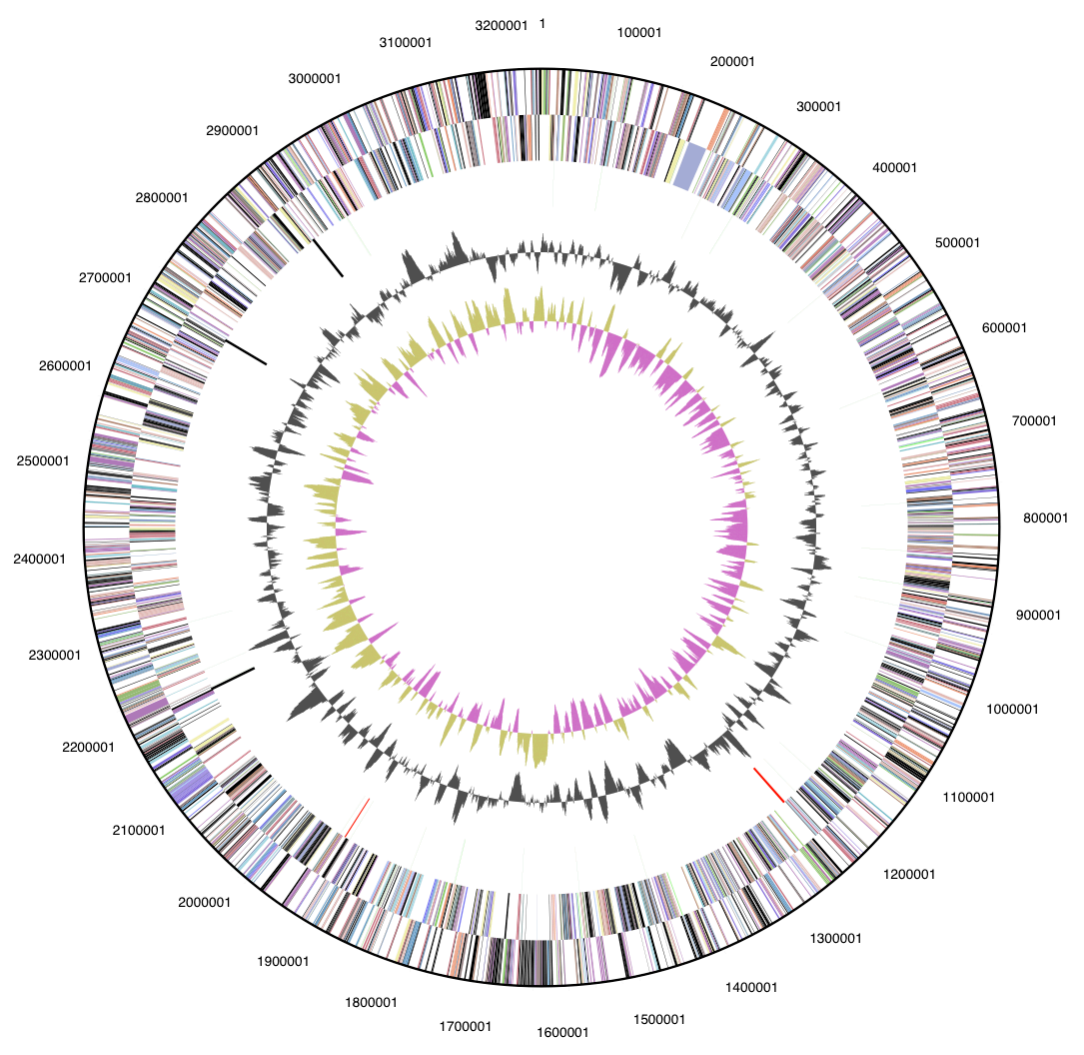
Graphical circular map of the genome. From outside to the center: Genes on forward strand (color by COG categories), Genes on reverse strand (color by COG categories), RNA genes (tRNAs green, rRNAs red, other RNAs black), GC content, GC skew.

The genome is 3,241,804 bp long and comprises one main circular chromosome with an overall GC content of 27.8% (Table 3 and Figure 3). Of the 2,893 genes predicted, 2,853 were protein-coding genes, and 40 RNAs. A total of 44 pseudogenes were identified. The majority of the protein-coding genes (66.2%) were assigned a putative function while those remaining were annotated as hypothetical proteins. The distribution of genes into COGs functional categories is presented in Table 4.

**Table 3:** Genome Statistics

**Attribute**	**Value**	**% of Total**
Genome size (bp)	3,241,804	100.00%
DNA coding region (bp)	2,841,470	87.65%
DNA G+C content (bp)	899,647	27.75%
Number of replicons	1	
Extrachromosomal elements	0	
Total genes	2,893	100.00%
RNA genes	40	1.38%
rRNA operons	1	
Protein-coding genes	2,893	98.62%
Pseudo genes	44	1.52%
Genes with function prediction	1,914	66.16%
Genes in paralog clusters	610	21.09%
Genes assigned to COGs	1,815	62.74%
Genes assigned Pfam domains	1,973	68.20%
Genes with signal peptides	577	19.94%
Genes with transmembrane helices	737	25.48%
CRISPR repeats	2	

**Table 4:** Number of genes associated with the general COG functional categories

**Code**	**value**	**%age**	**Description**
J	134	6.6	Translation, ribosomal structure and biogenesis
A	1	0.0	RNA processing and modification
K	81	4.0	Transcription
L	104	5.2	Replication, recombination and repair
B	0	0.0	Chromatin structure and dynamics
D	20	1.0	Cell cycle control, cell division, chromosome partitioning
Y	0	0.0	Nuclear structure
V	44	2.2	Defense mechanisms
T	116	5.8	Signal transduction mechanisms
M	143	7.1	Cell wall/membrane/envelope biogenesis
N	100	5.0	Cell motility
Z	0	0.0	Cytoskeleton
W	0	0.0	Extracellular structures
U	51	2.5	Intracellular trafficking secretion, and vesicular transport
O	62	3.1	Posttranslational modification, protein turnover, chaperones
C	111	5.5	Energy production and conversion
G	143	7.1	Carbohydrate transport and metabolism
E	185	9.2	Amino acid transport and metabolism
F	56	2.8	Nucleotide transport and metabolism
H	67	3.3	Coenzyme transport and metabolism
I	53	2.6	Lipid transport and metabolism
P	99	4.9	Inorganic ion transport and metabolism
Q	20	1.0	Secondary metabolites biosynthesis, transport and catabolism
R	286	14.2	General function prediction only
S	143	7.1	Function unknown
-	1,078	37.3	Not in COGs
